# An update on rehabilitative treatment of shoulder disease after breast cancer care

**DOI:** 10.1007/s12306-023-00806-w

**Published:** 2023-12-01

**Authors:** P. E. Ferrara, D. M. Gatto, S. Codazza, P. Zordan, G. Stefinlongo, M. Ariani, D. Coraci, G. Ronconi

**Affiliations:** 1 University Polyclinic Foundation A. Gemelli IRCCS, Rome, Italy; 2 Department of Neurosciences, Sense Organs and Thorax, Catholic University of the Sacred Heart, Rome, Italy; 3 Department of Neuroscience, Section of Rehabilitation, University of Padova, 35122 Padua, Italy

**Keywords:** Rehabilitation, Shoulder disease, Breast cancer, Disability

## Abstract

According to the latest statistics of the American Cancer Society 2022, breast cancer is a leading cause of morbidity and death among women worldwide. As a result of oncological procedures, breast cancer survivors often complain of pain and disability to the ipsilateral arm and shoulder. *Objective:* we aimed to analyze the latest literature regarding the efficacy of different rehabilitation treatments in patients affected by shoulder impairment secondary to breast cancer care. A comprehensive literature search was conducted on PubMed, PEDRO and Scopus databases. All English studies, published in the last decade up to March 2023, reporting shoulder problems in adult women treated for breast cancer with partial or total mastectomy ± breast reconstruction, lymphadenectomy, radio-, chemo-, hormonal or biologic therapy were assessed for eligibility. The methodological quality of the included trials was evaluated using the Cochrane bias tool. Of 159 articles identified, 26 were included in qualitative synthesis. Data from 1974 participants with a wide heterogeneity of breast cancer treatments were analyzed in this review. The methodological quality for most included studies was moderate. Several physiotherapy and interventional protocols showed some evidence of efficacy in shoulder range of motion (ROM), upper limb function, strength, pain and quality of life recovery after breast cancer treatment. Both physiotherapy alone or in combination with other techniques significantly improves shoulder disability, pain, and quality of life of patients undergoing breast cancer treatment regardless of their baseline characteristics or the time passed from surgery. The optimal treatment protocol and dosage remain unclear, and more homogeneous studies are needed in order to perform a meta-analysis of the literature.

## Introduction

Breast cancer is a leading cause of morbidity and death among women worldwide [1]; only about 1% of the breast neoplasms affects male individuals.

According to the “breast cancer statistics” of the American Cancer Society, in early 2022, more than 4 million of US women who received a previous breast cancer diagnosis were alive and the current survival estimates are 91% at 5 years and 84% at 10 years after the diagnosis [1]. As a result of oncological care, breast cancer survivors often complain of disabling side effects to the ipsilateral arm and shoulder such as pain and loss of function [2]. Physical disability may persist long-term after the treatments, significantly worsening the quality of life of these patients and limiting their ability to return to work [3, 4]. Based on the stage of the disease, breast cancer treatment may require one or more procedures such as surgery (both tumor resection and breast reconstruction), radiation therapy (RT), chemotherapy (CT), hormonal (HT) and nowadays also biologic therapies (BT) [3]. Surgical approach, which is the most frequent procedure, can be limited to the breast tissue alone or extend to the axillary lymph nodes. Although, in the last few decades, surgical techniques have become more and more conservative, in most cases, they still determine important tissue damage and side effects. Previous studies have found that patients treated with axillary lymph node dissection have the highest risk of developing impairment of the arm and shoulder, including range of motion (ROM) restriction, pain, lymphedema, reduced arm strength and limitation in daily life activities (ADLs) [3, 5–7]. Mastectomy does not directly damage the glenohumeral joint, however, post-surgical pain, antalgic postures, scar formation, and tension of the soft tissue can alter the shoulder girdle alignment and kinematic [8]. These biomechanical changes can lead to secondary pathologies such as bursitis, axillary cording, adhesive capsulitis, impingement, and myofascial pain that contribute to upper limb dysfunction [9].

Cancer rehabilitation helps patients to recover and maintain the highest physical, social, psychological, and vocational functioning within the limits created by the disease and its treatment [2]. Despite the high prevalence rate of breast cancer-related morbidity, different factors like poor clinician and patient knowledge about cancer rehabilitation, lack of clinician referral, motivational factors, economical barriers, and others, determine a marked underuse of these services [2]. In this review, we aimed to analyze the literature regarding the efficacy of different rehabilitation treatments in patients affected by shoulder impairment secondary to breast cancer care. We believe that this information is of direct clinical relevance as it may potentially reduce the risk of developing serious disability in the postoperative period and the associated healthcare and society costs.

## Materials and methods

### Search strategy

**Fig. 1:**
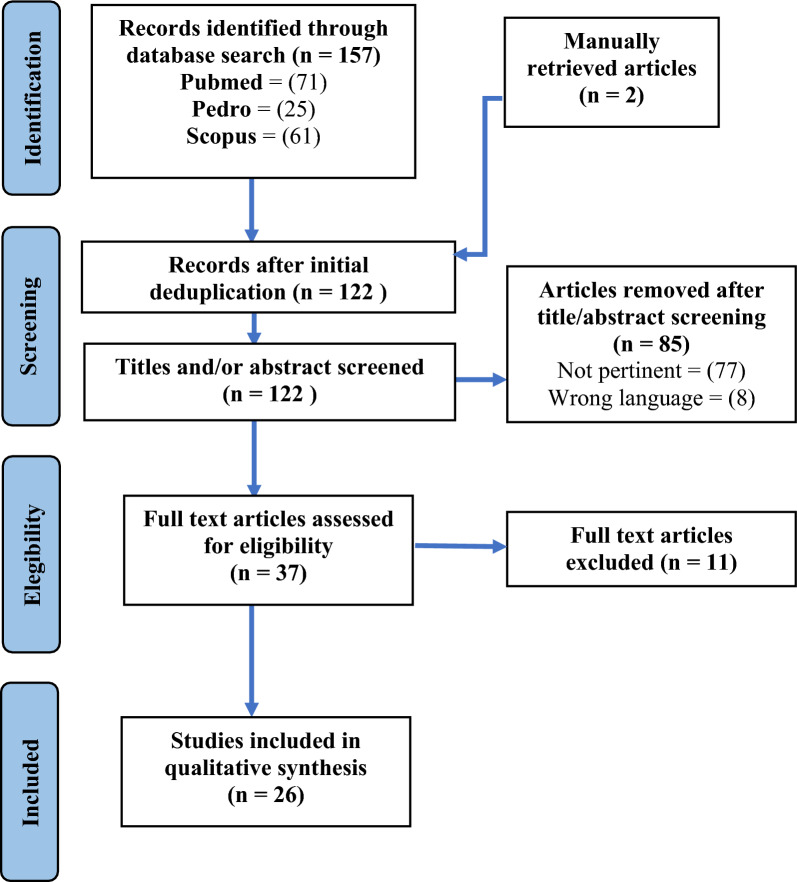
Study selection process

A comprehensive literature search was conducted on PubMed, PEDRO and Scopus databases using the following MESH terms: “breast cancer surgery” AND “shoulder” AND “treatment”. All English studies reporting shoulder problems in women treated for breast cancer with partial or total mastectomy ± breast reconstruction, lymphadenectomy, RT, CT, hormonal or biologic therapy were assessed for eligibility. Only randomized controlled trials (RCTs) or pseudo RCTs published in the last decade up to March 2023 were included. The literature search strategy is presented in Fig. 1.

### Study selection

Selection criteria for the studies were limited to adult women > 18 years treated for breast cancer with partial or total mastectomy ± breast reconstruction, lymphadenectomy, radio-, chemo-, hormonal or biologic therapy. Outcomes related to general upper limb function, limb strength, range of motion, quality of life and pain were extracted. All papers taking in consideration pediatric or male population, patients with previous shoulder trauma or surgery or papers focusing on lymphedema as exclusive shoulder problem were excluded. Eligibility criteria are summarized in Table 1.

**Table 1:** Study eligibility criteria

Criteria	Inclusion	Exclusion
Population	Adult women treated for breast cancer	Pediatric and male population
Intervention	Partial or total Mastectomy ± Lymphadenectomy, RT, CT, HT or BT	Previous shoulder trauma or surgery
Comparison	All treatment typology	
Outcome	ROM, QoL, Dash, Strength, VAS	Lymphedema as exclusive upper limb complication
Date	Last 10 years	
Language	English	

### Methodological quality

The methodology of this study was reported following the PRISMA Statement for systematic review and meta-analysis [10]. The Cochrane library assessment tool was used to evaluate the risk of bias in all the 29 selected studies according to the PRISMA guidelines [10]. A green light was assigned to a low risk of bias, a yellow light to an unclear risk of bias and a red light to a high risk of bias (Table 2).

**Table 2:**
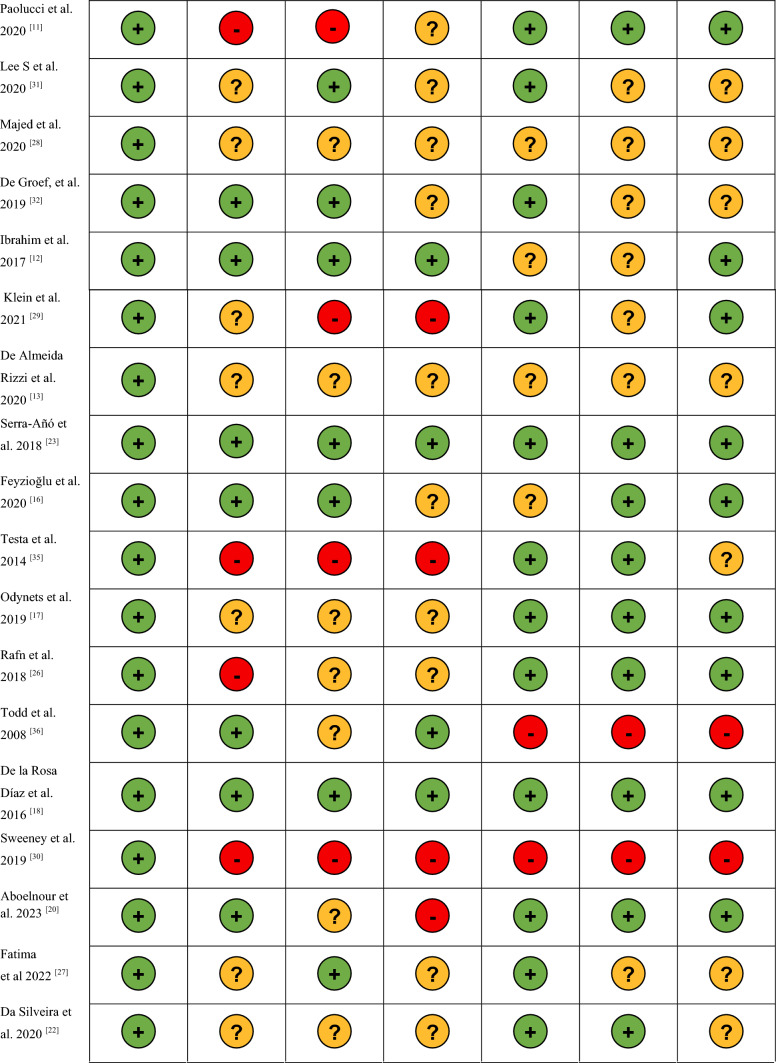

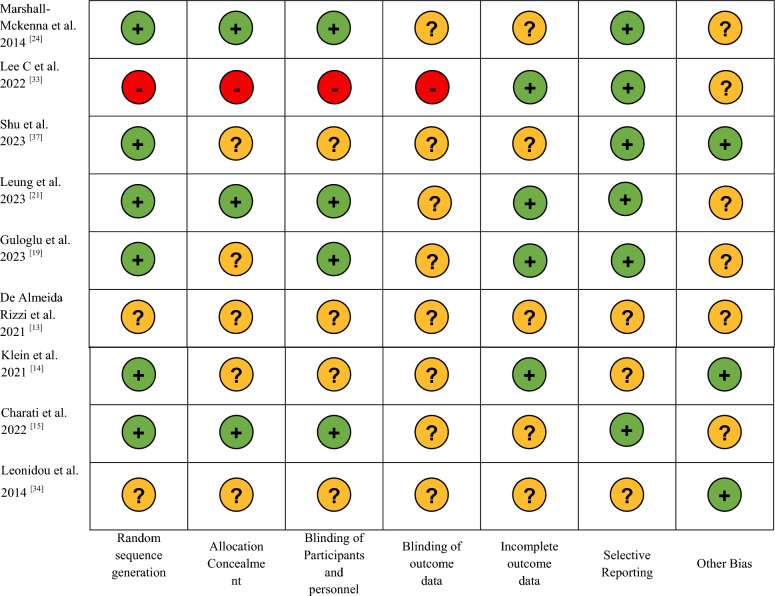
Evaluation of study bias

: Low risk bias

:High Risk of Bias

: Unclear Risk of

### Data extraction

To reduce the risk of inter-observer bias, two independent reviewers (P.Z., G.S.) selected the eligible articles for the review. In case of disagreement in the article selection or data extraction process a third author (P.E.F.) was involved. Data extracted from the selected studies were patients’ characteristics (number, mean age), breast cancer treatment, time since oncological care, type of shoulder problem, shoulder treatment protocol (experimental and control intervention), outcome measures, follow-up and main results (Table 3).

**Table 3:** Main results of the included studies

Article	Study group	Control group	Intervention	Outcome measures	Timing	Results
Lee C et al. 2022 [33]	n.23 BCS adhesive capsulitis Mean age (SD): 51.30 (± 6.58) Time from surgery: NR	n.44 idiopathic adhesive capsulitis Mean age (SD): 57.38 (8.53) Time from surgery: NR	Corticosteroid injection + home exercise training VS home exercise training	ROM, SPADI (only study group), capsular capacity, maximal pressure, capsular stiffness	T0: pretreatment T1: 2 weeks T2: 4 weeks	Significant higher maximal pressure capsular stiffness in study group Significant improvement in shoulder’s ROM and SPADI in the study group
Lee S et al. 2020 [31]	n.37 MAS, BCT, ALND Mean age (SD): 52.0 (± 9.8) Time from surgery: 0	n.46: MAS, BCT, ALND Mean age (SD): 51.3 (± 10.0) Time from surgery: 0	Guardix-SG® injection VS standard care	ROM, DASH, NRS, BIS	T0: baseline T1: 3 months T2: 6 months T3:12 months	No significant statistical differences between groups in ROM, DASH, VAS and BIS
De Groef et al. 2019 [32]	n.25 MAS, MAS + REC, BCT + pain at least from 3 months in pectoral region Mean age (SD):53.4 (± 10.0) Time from surgery:1.8 (± 1.6) years	n.25 MAS, MAS + REC, BCT + pain at least from 3 months in pectoral region Mean age (SD): 56.6 (± 10.0) Time from surgery:12.2 (± 2.3) years	Single BTX-A (100U) in pectoralis major muscle + standard physical therapy VS standard physical therapy	ROM, Upper limb strength, Scapular statics, DASH	T0 prior infiltration T1 1 month T2 3 months T2 6 months	No differences between groups in all outcome measures
Leonidou et al. 2014 [34]	n.7 BCS + RT + frozen shoulder secondary to breast cancer Mean age (SD): 48 yr Time from surgery: NR	n. 256 BCS + RT + non-BCS related frozen shoulder	Shoulder manipulation under anesthesia (MUA) + local corticosteroid injection VS standardized physiotherapy	OSS, ROM	NA	No difference between groups
Fatima et al. 2022 [27]	n.15 MAS + ALND Mean age (SD): NR Time from surgery: pre-operative	n.15 MAS + ALND Mean age (SD): NR Time from surgery: pre-operative	2–5 sessions per week of pre-operative stretching VS standard care	NRS, ROM, GARS	T0: 1st day post-surgery T1: 3rd day post-surgery	Significant improvement in pain, ROM (flexion, abduction) and, GARS in the study group
Byun et al. 2022 [25]	n.31 BCS, MAS Mean age (SD) 55.60 (± 12.41) Time from surgery: NA	n.30 BCS, MAS Mean age (SD) 56.16 (± 11.05) Time from surgery: NA	Printed education material + direct education VS Printed education materials	ROM, VAS, short DASH, SPADI, arm circumference	T0 before surgery T11 month T2 3 months T3 6 months T4 12 months after surgery	Significant improvement in the study group in short DASH, VAS and, SPADI 1 month after surgery and shoulder’s flexion/abduction up to 6 months post-surgery
Klein et al. 2021a [14]	n.72 BCT Mean age: 53.3 (± 12.7) Time from surgery: 1^st^ day	n.85 BCT Mean age: 51.2 (± 13.1) Time from surgery: 1^st^ day	Home-exercise instructions from post-op day 1 until ROM recovery VS standard care	NPRS, Quick DASH, ROM	T0: 1st day post-surgery T1: 1 month T2: 3 months T3: 6 months	Significant improvement in pain score at each follow-up and in quick-DASH at 6 months in the study group
Majed et al. 2020 [28]	n.30 MAS Mean age (SD): NR Time from surgery: 0	n.30 MAS Mean age: NR Time from surgery: 0	Pre-surgery education + demonstration of exercise VS standard care	ROM, Qol (Breast cancer version)	T0: pre-surgery T1: 2 weeks T2: 4 weeks after surgery	Significant improvement in ROM flexion, extension, abduction and Qol in the study group at T1 and T2
Rafn et al. 2018 [26]	n: 21 BCS, MAS, ALND Mean age (SD): 55.05 (± 6.4) Time from surgery: pre- and immediately post-surgery	n:20 BCS, MAS, ALND Mean age (SD): 53.25 (± 10) Time from surgery: pre- and immediately post-surgery	Pre-surgery education for both groups Prospective surveillance and targeted physiotherapy (PSTP) VS education only (EDU)	ROM, arm strength, arm volume, NRS, Q-DASH, FACIT-B	T0: pre-surgery T1: 12 weeks after surgery	n.10 PSTP and n.8 EDU patients had arm morbidity at T1 with more complex arm morbidity in EDU group
Leung et al. 2023 [21]	n.54 MAS Mean age (SD): 57.4 (± 11.7) Time from surgery: 6 weeks	n.54 MAS Mean age (SD): 61.8(± 12.7) Time from surgery: 6 weeks	2x/week for 6 weeks of conventional physiotherapy + mechanical surgical scar stimulation VS conventional physiotherapy	VSS, DASH, Shoulder ROM, Hand grip strength, FACT-B	T0: baseline T1: after treatment T2: 3 months T3: 6 months	Significant improvement in VSS, DASH and FACT-B in the study group
Guloglu et al. 2023 [19] *Only abstract available	n. 22 ALND + RT Mean age (SD): 46.0(7.7) Time from RT: 19.2 11.8	n. 22 ALND + RT Mean age (SD): 44.2(7.0) Time from RT: 19.4 (11.6)	Proprioceptive Neuromuscular Facilitation (PNF) VS Progressive resistance training (PRT)	VAS, upper extremity strength, DASH, GRCS	T0: baseline T1: 8 weeks after treatment	Significant improvement in within analysis in both PNF and PRT groups
Aboelnour et al. 2023 [20]	n. 35 post-MAS adhesive capsulitis Mean age (SD): 49.54 (± 5.33) Time from surgery:12.57 (± 1.68)	N:35 post-MAS adhesive capsulitis Mean age (SD): 50.57(± 4.69) Time from surgery: 13.05 (± 1.59)	5x/week for 8 weeks Thera-band exercise + standard care VS standard care	Shoulder ROM, grip strength, DASH, VAS, SF-36	T0: baseline prior treatment T1: 8 weeks	Significant improvement in all outcome measure in the study group
Shu et al. 2023 [37] *Only abstract available	200 ALND patients **A group**: ROM training 7postoperative day + PRT at 4 weeks **B group**: ROM training 7postoperative day + PRT at 3 weeks **C group**: ROM training 3postoperative day + PRT at 4 weeks **D group**: ROM training 3 postoperative day + PRT at 3 weeks		–	CMS, Shoulder ROM and strength, Grip, EORTC QLQ-BR23, SF-36	–	Significant better ROM CMS and, quality of life in group C and D
Charati et al. 2022 [15]	n. 35 BCS Mean age (SD): NR Time from surgery: NR	n. 35 BCS Mean age (SD): NR Time from surgery: NR	Motor exercises (stretching, aerobic) for 5 weeks after surgery VS Standard care	Shoulder ROM, 6MWT, EORTC QLQ-C30 and QLQ-BR23, HADS	T0: before surgery T1: 2 days T2: 5 weeks after surgery	Significant improvement in 6MWT, shoulder ROM, depression and anxiety in the study group
De Almeida Rizzi et al. 2021 [13]	n. 30 BCS Mean age (SD): NR Time from surgery: day 1 post-surgery	n. 30 BCS Mean age (SD): NR Time from surgery: day 1 post-surgery	Week 0–2 post-surgery: exercises limited to 90° for both groups From week 2 on, patients’ randomization into: 90°-limited ROM group VS free ROM group	N° of complications, ROM, Pain, Upper limb function	–	No differences between groups in all outcome measures
Klein et al. 2021b [29]	n. 107 BCT Mean age (SD): 51.4 (± 12.4) Time from surgery: NR	n. 50 BCT Mean age (SD): 53.8 (± 13.8) Time from surgery: NR	Low, moderate and high intensity physical activity (PA) group VS inactive group	NPRS, Quick DASH, ROM, Self-efficacy Return to job status, Number of sick days, adverse reactions	T0: 1 month T1: 3 months T2: 6 months	Better ROM, self-efficacy, return to prior job status and lower disability in higher PA groups. PA level did not influence pain at any time. High PA increased the risk of axillary web syndrome
Paolucci et al. 2020 [11]	n. 29 MAS Mean age (SD): 51.6(± 9,8) Time from surgery: 10.36 (± 4.03) months	n.30 BCT Mean age (SD): 53.17(± 10,6) Time from surgery: 10.80 (± 6.0) months	2x/week for 4 weeks (12 sessions) of “Integrated low intensity rehab intervention”	DASH, VAS, BTS reaching tasks	T0: surgery T1: 6 weeks T2: 3 months	Improved VAS in within-group analysis in both groups
Da Silveira et al. 2020[22]	n. 20 BCS + ALND Mean age (SD): 52.2(± 8.31) Time from surgery: NR	n.12 BCS + ALND Mean age (SD): 48.4(± 1.12) Time from surgery: NR	Standard care + PNF (3x/week for 4 weeks) VS Standard care	Shoulder ROM, grip strength, lymphatic circulation analysis	T0: 1 day before treatment T1: after treatment	Significant improvement in ROM flexion, abduction, external rotation and, grip strength in the study group
Feyzioğlu et al. 2020 [16]	n. 20 MAS + adjuvant therapy Mean age (SD): 50.84(± 8.53) Time from surgery: NR	N:20 MAS + adjuvant therapy Mean age (SD): 51.0(± 7.06) Time from surgery: NR	Weeks 0–2: both groups standard physiotherapy Weeks 2–6: Study group Xbox Kinect™ (45 min 2x/week for 4 weeks) VS standard physiotherapy	VAS, ROM DASH, grip strength, upper limb muscle strength, TKS	T0: before treatment T1: after treatment	Significant improvement in TKS in the study group. No difference between groups in VAS ROM, hand grip and muscle strength
Odynets et al. 2019 [17]	n.34 MAS Mean age (SD): 57.44 (± 2.16) Time from surgery: < 6 months	n. 34 MAS Mean age (SD): 57.99 (± 2.24) Time from surgery: < 6 months	3x/week for 3 months of water exercises VS pilates	Shoulder ROM, Upper limb strength and arm circumference	T0: before exercises T1: 12 weeks after exercises	Significant better ROM flexion and abduction and less lymphedema in the study group
Sweeney et al. 2019 [30]	n. 50 BCS + ALND + adjuvant therapy + obesity (BMI at least 25.0 kg/m2) Mean age (SD): 52.8 (± 10.6) Time from surgery: < 6 months	n. 50 BCS + ALND + adjuvant therapy + obesity (BMI at least 25.0 kg/m2) Mean age (SD): 53.6 (± 10.1) Time from surgery: < 6 month	Exercise (aerobic and resistance training) 3x/week for 16 weeks VS Usual care	Shoulder ROM, muscular strength, DASH	T0: baseline T1: 4 months T3: 3 months only for study group	Significant improvement in muscular strength, ROM, and DASH in study group
Serra-Añó et al. 2018 [23]	n.13 BCS Mean age (SD): 53.15(± 10.91) Time from surgery: at least 4 months earlier	n.11 BCS Mean age (SD): 54.36 (± 6.86) Time from surgery: at least 4 months earlier	Myofascial release (1x/week for 4 weeks) VS manual lymphatic drainage	VAS, ROM, DASH, PHQ-9	T0: before treatment T1: after treatment T2: 1 month later	Significant better ROM (except external rotation), DASH and Qol in the study group
Ibrahim et al. 2017 [12]	n.29 BCT + RT Mean age (SD): NR Time from surgery: NR	n.30 BCT + RT Mean age (SD): NR Time from surgery: NR	Progressive physical therapy (2x/week for 12-weeks) VS standard care	ROM and Pain	T1: pre-RT T2: post-RT T3: 3 months T4: 6 months T5: 12 months T6: 18 months post-RT	Better ROM and pain scores 12 weeks post-radiation in the study group No differences between groups at 18 months
De la Rosa Díaz et al. 2016 [18]	n.10 BCS + ALND + adjuvant therapy Mean age (SD): 54.8 (± 15.6) Time from surgery: 3rd and 6th day after surgery	n.8 BCS + ALND + adjuvant therapy Mean age (SD): 45.9(± 6.2) Time from surgery: 3rd and 6th day after surgery	Accessory joint mobilization VS Neural mobilization (30 min-sessions, 3x/week for 3 weeks)	Shoulder ROM, VAS, Wingate Daily Life Activities Table	T0: prior surgery T1: 3–6 days after surgery T2: after treatment T3: 3 months T4: 6 months	Within groups improvement in shoulder ROM flexion and abduction at T2 (Preliminary results)
Testa et al. 2014 [35]	n. 35 BCS + MAS + ALND Mean age (SD): 54.3 (± 8.02) Time from surgery < 1 week	N:35 BCS + MAS + ALND Mean age (SD): 55.3 (± 8.5) Time from surgery < 1 week	Cautious arm mobilization on day 2–5 post-surgery From 5th day on, physiotherapy 5x/week for 4 weeks VS standard care	EORTC QLQ-30, EORTC QLQ-BR23, ROM	T0: before surgery T1: 5th day after surgery T2: 1 month T3: 6 months T4: 12 months	Better Qol and ROM at T4 and lower pain at each time point in the study group
Marshall-Mckenna et al. 2014 [24]	n.14 BCS + RT Mean age (SD): 63.5 (± 11.1) Time from surgery: NR	N:10 BCS + RT Mean age (SD): 51.4 (± 11.9) Time from surgery: NR	Myofascial release (20–30 min-sessions 1x/week for 3 weeks) VS Usual care	Shoulder ROM, MPQ, DASH, HADS	T0: 1 day before treatment T1: 4 weeks	Significant improvement in shoulder ROM in the study group
Todd et al. 2008 [36]	n. 58 MAS + ALND + adjuvant therapy Mean age (SD): 57.2(14) Time from surgery: post-surgery	n. 58 MAS + ALND + adjuvant therapy Mean age (SD): 56.5 (± 12.4) Time from surgery: post-surgery	Delayed arm mobilization (7 days after surgery) VS Early arm mobilization (within 48 h from surgery) with vigorous exercises and mobilization until 90° after surgery	Limb volume, Grip strength, ROM, FACTB + 4, SDQ	T0: pre-surgery T1: 12 weeks after surgery	Lower rate of lymphedema in the study group vs control group at T1 (N:16 in study group vs N: 6 in control group). No difference between groups in other outcome measures

*ALND* axillar lymph node dissection; *ALND* axillar lymph node dissection; *BCS* breast cancer surgery; *BCT* breast conservative treatment; *BIS* Bioelectrical impedance spectroscopy; *CMS* Constant-Murley score; *DASH* Disabilities of the arm shoulder and hand questionnaire; *EORTC QLQ-BR23* European Organisation for Research and Treatment of Cancer; *FACTB* functional assessment of chronic illness therapy–breast cancer; *GARS* Groningen Activity Restriction Scale; *GRCS* global rating of change scale; *HADS* hospital anxiety and depression scale; *KBRG* kinect-based rehabilitation group; *LA* local anesthetic; *MAS* mastectomy; *MPQ* McGill pain questionnaire; *MUA* manipulation under anesthesia; *NA* not applicable; *NPRS* numeric pain rating scale; *NR* not reported; *OSS* Oxford shoulder score; *PHQ-9* pain health questionnaire-9; *PNF* proprioceptive neuromuscular facilitation; *PPP* persistent postoperative pain; *PRT* progressive resistance training; *PSS* Penn shoulder scale; *REC* reconstruction; *ROM* range of motion; *SDQ* shoulder disability questionnaire; *SF-36* Short Form Health Survey 36; *SPADI* shoulder pain and disability index; *SPTG* Standard Physiotherapy Group; *TKS* Tampa Kinesiophobia scale; *TPVB* thoracic paravertebral block; *VAS* visual analogue scale; *VSS* Vancouver scar scale; *NPRS* numeric pain rating scale

## Results

### Descriptive analysis

The search was conducted on PEDRO, PubMed, and Scopus search engines and identified 159 articles. After removal of duplicates, 122 articles were reviewed by title and abstract, 37 full-text articles were assessed for eligibility, and 26 studies were finally included in qualitative synthesis.

### Patients’ characteristics

A total of 1974 participants were included in this systematic review with a wide heterogeneity of breast cancer treatments (mastectomy or breast conserving surgery, neoadjuvant/adjuvant chemotherapy and/or radiotherapy, lymph node dissection or a combination of these types of treatments). All the patients were female, as per inclusion criteria; the mean age was 53.47 years and 52.9 years for the experimental group and the control group, respectively. The main results of each selected paper are summarized in Table 3.

### Study design

One study investigated conventional physiotherapy (CP) in patients operated of mastectomy versus quadrantectomy [11]. Four studies [12–15] investigated a specific physiotherapy program versus standard care. Four studies compared two individualized physical interventions such as conventional physiotherapy versus Xbox 360 Kinect-based virtual reality training [16], pilates versus water-based exercises [17], accessory joint mobilization (AJM) versus neural mobilizations (NM) [18] and proprioceptive neuromuscular facilitation (PNF) versus progressive resistance training (PRT) [19]. Three studies investigated conventional physiotherapy alone or in addition with Thera‐Band exercises [20], mechanical stimulation of mastectomy scar [21] or proprioceptive neuromuscular facilitation (PNF) [22]. Two studies investigated the effects of myofascial release [23, 24]. Four studies investigated the effects of preoperative interventions on postoperative outcome, such as patients’ education [25, 26], stretching [27] and extensive pre-surgery physiotherapy [28]. In terms of exercise intensity, two study investigated the effects of different level of physical activity on different post-surgical outcomes [29, 30]. Other studies investigated several interventional treatments such as the administration of a mixture of poloxamer and sodium alginate (Guardix-SG®) after axillary lymph node dissection versus no treatment [31] and Botulinum Toxin A injection versus placebo saline solution [32]. Three studies specifically focused on cancer related adhesive capsulitis: Aboelnour et al. [20] compared physiotherapy alone or in combination with Thera-band exercise while Lee C. et al. [33] and Leonidou et al. [34], compared hydrodilatation with corticosteroids injection and manipulation under anesthesia, respectively, for adhesive capsulitis of different etiology.

### Methodological quality

Except for Serra-Añó et al. [23], Ibrahim et al. [12], De la Rosa Díaz et al. [18], all the included studies are of moderate quality. Most of the studies have a moderate risk of bias in allocation concealment and blinding of participants, personnel and outcomes (Table 2). Only two studies show high methodological risk of bias [30, 33].

### Outcome measures

Almost all the studies performed goniometric analysis of the shoulder ROM, Paolucci et al. [11] also included a biomechanical evaluation during reaching tasks and De Groef et al. [32] a scapular static and kinematic measurement. The visual analogue scale (VAS) [11, 16, 18–20, 23, 25, 35], Numerical Pain Rating Scale (NRS or NPRS) [14, 21, 26, 27, 29, 31], Constant–Murley Score, McGill Pain (MPQ) [24] and the Oxford shoulder score (OSS) [34] were used for pain evaluation. Regarding upper limb disability most studies used the DASH scale, four studies [14, 25, 26, 29] the QuickDASH, one study [18] the Wingate Daily Life Activities Table and one study [27] the Groningen Activity Restriction Scale (GARS). Quality of life parameters have been also extensively studied, Majed et al. [28] used the Quality-of-life Breast Cancer Patient Version scale (QoL-BC), five studies used [21, 23, 26, 36] the Functional Assessment of Cancer Therapy for breast cancer patients (FACT-B + 4), two studies [15, 35] the EORTC QLQ-30 questionnaire by, three studies [15, 35, 37] the EORTC QLQ-BR23 questionnaires, and two studies [20, 37] the Short Form Health Survey 36 (SF-36) (Table 3).

### Timing of rehabilitation from breast cancer treatment

Regarding the time to begin rehabilitation since breast cancer treatment, six studies begun the intervention preoperatively [24–29], seven studies within 1 week [13, 14, 16, 31, 33, 35, 36], two studies between the second week and 3 months [12, 32] and four studies after more than 3 months [11, 17, 23, 30]. Finally, nine studies [13, 15, 18–22, 34, 37] did not specify the beginning of the treatment protocol, or the information was not available from the abstract.

### Exercise type, frequency, and intensity

Details about exercise protocols are displayed in Table 3.

## Discussion

The objective of this review was to provide an overview on the current state of the literature regarding all possible rehabilitative and interventional treatments for shoulder disease following breast cancer surgery. We found 26 RCTs focusing on the treatment of shoulder pathology after breast cancer care; however, only very few studies described a specific shoulder pathology (i.e., adhesive capsulitis), while most of them focused on generic shoulder impairment. Furthermore, a very high heterogeneity in the literature was observed regarding treatment choice and protocol used (timing of rehabilitation, frequency, intensity, outcome measures and follow-up). Here, we provide a summary of the evidence.

### Type of treatment

Four studies evaluated the effect of physical therapy on different disability and quality of life outcomes. Ibrahim et al. [12] did not find a statistically significant improvement in pain and shoulder ROM with a 12-week program of progressive upper limb mobility, strength and endurance exercises versus standard care. Conversely, Charati et al. [15] found a statistically significant increase in shoulder ROM, 6-min walking test scores, QoL, depression and anxiety after 5 weeks of stretching and aerobic exercises compared to standard hospital care. In these two studies the patients underwent a similar oncological and rehabilitation treatments, however in the study of Charati et al. [15] the patients started rehabilitation before surgery. The study of Paolucci et al. [11] found improvement in pain and upper limb function but without significant difference between women operated of mastectomy and quadrantectomy. Although more invasive techniques are typically associated with worse outcomes [3], in this case, both patients’ groups begun physiotherapy long after the surgical procedure (**~ **10 months) when they had similar baseline VAS and DASH scores. A study of Guloglu et al. [19] found that both proprioceptive neuromuscular facilitation (PNF) and positional release technique (PRT) resulted in a statistically significant change in shoulder ROM, strength, power, endurance, pain, and functionality. However, PNF resulted the most effective technique in terms of functional recovery.

When comparing physiotherapy alone or in combination with other treatments, Aboelnour et al. [20] found that progressive graded Thera-Band exercises and scapular stabilization exercises in addition to conventional physiotherapy was superior then PT alone at improving shoulder VAS, DASH, ROM, limb strength and QoL in patients affected by secondary adhesive capsulitis. Leung et al. [21] found that adding mechanical massage of the mastectomy scar to physiotherapy was superior than physiotherapy alone in terms of DASH and QoL scores. Finally, da Silveira et al. [22], showed that the combination of standard physiotherapy and proprioceptive neuromuscular facilitation was superior to single physiotherapy in terms of palmar grip strength, shoulder ROM but it didn’t influence lymphatic flow.

Two studies compared conventional physiotherapy against different typology of treatments. Feyzioğlu and colleagues [16] found a comparable improvement in pain, shoulder ROM, muscle strength, grip strength and Tampa Kinesiophobia Scale scores after 6-week of conventional physiotherapy versus Xbox 360 Kinect-based rehabilitation program. However, standard physiotherapy appeared to be more effective in terms of upper limb functional recovery compared to virtual training. This is the only study applying virtual reality and gaming to breast cancer rehabilitation. We believe that this technique might be further investigated in the near future. Odynets et al. [17] found that a 12-week program of water-based exercise was better than pilates for active shoulder ROM and upper limb lymphedema but did not report significant differences in upper limb strength between the two groups. Water therapies have been long studied for the treatment of different pathologies including cancer related symptoms [38]. The capacity of cold water to induce peripheral vasoconstriction followed by vasodilatation as well as the massaging effect have been used to help alleviate lymphedema. Warm water instead comes with analgesic and antiphlogistic effects. Furthermore, the hydrostatic pressure of the water and the buoyancy are exploited during exercise therapy to improve muscle stretching, body strength and mobility in in people with musculoskeletal and neurological disorders [38]. On the other side, pilates combines physical exercise with mindfulness; this has been shown to improve individual’s body and kinesthetic awareness thus having the potential to improve both physical and psychological aspects of breast cancer patients [39].

Three studies investigated myofascial therapy for shoulder problems in breast cancer survivors. Serra-Añó et al. [23] found that 4 weeks of myofascial release treatment was better than a placebo manual lymphatic drainage in terms of pain, shoulder ROM, functionality, QoL and physical well-being. Similarly, Marshall-Mckenna et al. [24] demonstrated a statistically significant efficacy of myofascial release treatment in shoulder ROM compared to no physiotherapy. However, no significant difference in pain, functionality, anxiety, or depression were found between the two groups in the follow-up period. De la Rosa Díaz et al. [18] compared accessory joint mobilization (AJM) vs neural mobilization (NM) techniques for shoulder motion restriction and found a higher recovery in shoulder flexion and abduction with AJM. These results are in line with the literature. A recent review of Kalichman et al. [7] on myofascial therapy in breast cancer suggests that myofascial release techniques are efficacious in reducing the prevalence of active myofascial trigger points and therefore decrease pain sensitivity and improve ROM.

Some experimental studies explored some “interventional type” treatments for shoulder problems after breast cancer care. Lee S et al. [31] found a non-statistically significant effect of an anti-adhesion agent consisting of poloxamer and sodium alginate (Guardix-SG®) for the prevention of upper extremity dysfunction after ALND. De Groef et al. [32] found improvement of persistent upper limb pain after a single Botulinum Toxin A infiltration in the pectoralis major muscle in addition to a standard physical therapy, however, no significant differences in upper limb function and QoL were found between the groups.

Three studies focused on a specific shoulder pathology (i.e., adhesive capsulitis): Aboelnour et al. [20] found that physiotherapy in addition to thera-band exercises was more effective than single physiotherapy in terms of VAS and DASH scores, shoulder ROM, strength and QoL. Lee C. et al. [33] tested the effect of hydrodilatation + corticosteroid injection + home exercise training in patients with adhesive capsulitis secondary to breast cancer surgery versus primary adhesive capsulitis and found a more significant improvement in shoulder ROM, SPADI scores and disability sub-scores in the breast cancer related AC group. Leonidou et al. [34] compared the effect of manipulation under anesthesia, injection of local anesthetic and corticosteroid and physiotherapy and found a comparable improvement in shoulder ROM and Oxford Shoulder Scale scores in a group of adhesive capsulitis secondary to breast cancer treatment versus a non-cancer-related adhesive capsulitis group. Adhesive capsulitis is a condition characterized by an insidious onset of shoulder pain and multidirectional ROM limitation. According to Yang et al. [8], mastectomy is a major risk factor for its development, and breast reconstruction additionally increases this risk compared to breast sparing surgery.

In one of the three study the patients did actually undergo radical mastectomy [20], while in all of them, most of the patients were subjected to RT. A study of Yang et al. [8] found evidence that radio- and hormonal therapy constitute a major risk factor for pain and fibrosis of the shoulder and chest wall [2, 3]. Likewise, a study of Højris et al. [40] found a significantly higher incidence of shoulder morbidity in women treated with postsurgical radiotherapy compared to controls [40]. Irradiation induces an overproduction of proinflammatory mediators. Locally released cytokines participate in several physiological responses, including pain response and tissue fibrosis [4]. Intraarticular corticosteroid injection and stretching exercises confer rapid pain relief and helps the recovery of ROM in early-phase adhesive capsulitis [8]. Further studies are needed to investigate how the different types of adhesive capsulitis respond to treatment.

### Timing of rehabilitation and postoperative complications

Early physiotherapy was found to have a variable effect in several studies. In the study of Klein et al. [14], physiotherapy commenced early from post-op day 1 reduced pain levels at 1 and 6 months of follow-up compared with controls who did not receive physiotherapy. The rehabilitation protocol did not improve the participants' flexion and abduction nor influenced the incidence of postoperative complications. Conversely, Todd et al. [36] found a significantly higher incidence of lymphoedema in women who underwent full shoulder mobilization within 48 h. After analyzing the two studies, we noticed a difference in the characteristics of the population sample. In Todd et al. [36], the patients underwent a more invasive type of surgery (i.e., mastectomy + axillary lymphadenectomy) which could explain the difference in the rate of incidence of lymphedema compared to Klein et al. [14]. In Testa et al. [35], the control group received early physiotherapy from post-op day two. Compared with the control group, the treatment group showed reduced pain at 1 month and up to 12 month and improved glenohumeral joint mobility and QoL. De Almeida Rizzi [13] randomized 60 women treated with mastectomy and immediate breast reconstruction into a ‘‘free-range group’’ with shoulder range exercises performed until limited by pain or wound dehiscence and a ‘‘limited-range group’’ with movement restriction at 90° until 30 days after surgery. Both groups started physiotherapy 48 h after surgery and did not differ in terms of incidence and prevalence of postoperative complications. Patients in free-range group, however, had less pain, greater shoulder amplitude, and better upper limb function than the counterpart. Shu et al. [37] which involved 200 breast cancer patients who underwent axillary lymph node dissection, randomly assigned them into 4 groups starting ROM training at 3 or 7 days and progressive resistance training (PRT) at 3 or 4 weeks postoperative. Participants who started ROM training at 3 days and PRT at 3 weeks postoperative had more benefits in mobility, shoulder function and strength and quality of life scores, respectively. Incidence of adverse reactions was low in all 4 groups, with no significant differences among them. Based on this evidence, it seems reasonable to limit shoulder ROM exercise below 90° for the first 48 h after surgery in women who underwent more invasive procedure (i.e., mastectomy with axillary lymph node dissection).

### Frequency and intensity of rehabilitation

The study of Klein et al. 2021b [29] found that high preoperative intensity and frequency of activity (> 5 h per week) was associated with higher postoperative functional improvement, self-efficacy and percentage of return to preoperative job and reduced risk of chronic pain compared with inactive a low-active individuals. Moreover, patients in the inactive group received on average more neoadjuvant and radiation treatments compared with the active group. No effect on exercise intensity was found on the incidence of seroma, infections, and lymphedema; however, vigorous PA level was associated with an increased risk of axillary web syndrome. The RCT of Sweeney et al. [30] found that 4-month progressive combined aerobic and resistance exercise intervention (150 min of aerobic exercise and 2–3 days of resistance exercise training per week) against routine physical activity significantly increased active ROM, strength and disability versus routine activity. The exiguity and heterogeneity of protocols that focus on frequency and intensity of training did not allow for an accurate comparison between studies; however, it seems that more intense programs lead to better outcomes. More studies are needed to clarify which spectrum of frequency and intensity best suits for women undergoing oncologic treatment.

## Limitations

There are several limitations that must be noted. First, most of the studies reported functional scores without a proper characterization of the underlying causative condition. Indeed, none of the study included a radiological investigation of the shoulder. Second, the vast heterogeneity of protocols and outcome measures used did not allow for a comparison of treatments between studies. Third, the temporal limitation to the last 10 years might have excluded other good quality RCTs. Fourth, some studies had a small sample size.

## Conclusions

Through our review, it certainly emerged that physical therapy improves shoulder disability, pain, and QoL of patients undergoing breast cancer treatment regardless of the baseline characteristics of the patients or the time passed from surgery. The optimal treatment protocol and dosage remain unclear, and more homogeneous studies are needed to perform a meta-analysis of the literature. The analysis of the literature has also highlighted how the diagnostic component is still very lacking and many shoulder impairments are treated without a specific characterization of the underlying disease. This is a major limiting factor to the standardization of rehabilitation protocols and reduces the specificity of the treatments.

### Take home message

- Physical therapy improves shoulder disability, pain, and Qol of patients undergoing breast cancer treatment regardless of the baseline characteristics of the patients or the time passed from surgery.
- Physiotherapy in addition with other techniques such as Thera‐Band exercises, mechanical stimulation of mastectomy scar, proprioceptive neuromuscular facilitation and myofascial therapy has shown to be superior than physiotherapy alone.
- There is still too few evidence in favor of the use of virtual reality training versus conventional physiotherapy, water-based exercises versus pilates, accessory joint mobilization versus neural mobilizations or botulinum toxin A injections in the pectoralis muscle for pain.
- Is not recommended, at the moment, the administration of poloxamer and sodium alginate (Guardix-SG®) after axillary lymph node dissection.
- Preoperative patient's education and physiotherapy lead to better post-treatment outcomes.
- In order to reduce the occurrence of seroma and lymphedema, shoulder ROM exercise above shoulder’s level should be limited for the first 48 h, especially in women who underwent more extensive surgical interventions.
- In terms of exercise frequency and intensity, there is some evidence that high physical activity after surgery leads to better outcomes; however, the optimal dosage remains unclear.
